# Brown Adipocyte Promotes HR+ Breast Cancer Invasiveness Through IRX3-Mediated Mitochondrial Dysfunction

**DOI:** 10.3390/metabo16060349

**Published:** 2026-05-22

**Authors:** Shihang Hu, Bin Hu, Shiqiong Su, Ying Zhou, Gang Liu, Yuzhe Gao, Qing Ni, Jing Hou

**Affiliations:** 1 Department of Breast Surgery, Guizhou Provincial People’s Hospital, Guiyang 550002, China13985527762@163.com (Q.N.); 2 Graduate School, Guizhou Medical University, Guiyang 550004, China

**Keywords:** breast cancer, brown adipocytes, IRX3, mitochondria

## Abstract

**Background:** Adipocytes play a critical role in the breast cancer tumorigenic microenvironment. However, their effects and underlying mechanisms remain unclear. This study aims to investigate the role of adipocytes in luminal A breast cancer invasiveness at the cellular and molecular levels. **Methods:** Various adipocyte types were co-cultured with MCF7 breast cancer cells in direct and indirect manners. Invasiveness was assessed via proliferation, migration, and invasion, with alterations examined at morphological, cellular, and molecular levels. The role of adipocytes on MCF7 was further explored using an orthotopic breast cancer xenograft mouse model. **Results:** MCF7 co-cultured with adipocytes, especially brown adipocytes (BAC), showed increased invasiveness and tumorigenic potential. Morphologically, co-cultivation with BAC increased the proliferation, EMT, and stemness of MCF7. Mechanistically, co-culture of MCF7 with BAC exhibited disturbed expression of genes related to adipogenesis and mitochondrial dynamics; notably, IRX3 was the most prominently elevated one. Knockdown of IRX3 restored balanced mitochondrial function and reduced both the invasiveness of breast cancer cells in vitro and tumor growth in vivo. **Conclusions:** Brown adipocytes promote breast cancer invasiveness by upregulating adipogenesis-related IRX3, which acts via the mitochondrial functional regulation.

## 1. Introduction

Surgical resection remains the primary therapeutic approach for breast cancer, and is advancing towards higher cure rates, a minimally invasive approach, and a new era of post-operative breast reconstruction [1]. Autologous tissue breast reconstruction is the most commonly used reconstruction approach; however, it is associated with larger trauma, longer hospitalization, and postoperative complications such as swelling, thrombotic skin flap necrosis, and weakened abdominal wall [2]. As a new technique of breast reconstruction, autologous fat transplantation (AFT) has the advantages of a simple procedure, good appearance, excellent radiation permeability and tolerance, as well as sufficient tissue source, leading to its growing clinical adoption [3]. However, the application of AFT in patients with breast cancer has always been controversial due to concerns regarding oncological safety, especially with the introduction of adipose-derived stem cell (ADSC)-assisted fat transfer [4,5]. Numerous clinical studies have shown no significant increase in local recurrence rate (LRR) for breast cancer patients receiving AFT compared to patients without AFT. The only difference was recently demonstrated in luminal A molecular subtype patients after AFT, which showed that the LRR increased significantly in the 10-year follow-up [6]. However, the roles of different types of adipocytes in luminal A breast cancer recurrence were not determined. Specifically, at the cellular level, concerns about the risk of postoperative cancer recurrence originate from the hypothesis that the interplay between transplanted adipose tissue and dormant breast cancer cells would remodel the breast cancer microenvironment. However, the underlying mechanisms remain unclear. Therefore, in this study, we will investigate the phenotype and mechanisms of various adipocytes on the luminal A subtype breast cancer invasiveness.

Due to the unique anatomical structure of adipose and breast gland tissue wrapping around each other, it is obvious that adipocytes and glandular epithelial cells, as well as cancer cells, influence each other [7]. Moreover, as a complex endocrine organ, adipose tissue plays a critical role in obesity and cancer, a relationship that has been extensively studied. Adipocytes are classified based on their origin and characteristics, including ADSC, immature preadipocytes, as well as mature white and brown adipocytes (WAC and BAC). In terms of the composition of mammary adipose tissue (MAT), breast adipose tissue also consists of various types of adipocytes, abundant in mature WAC, with a decreasing content of BAC with age [8,9]. In addition, it was reported that ADSCs and progenitor cells from the stromal vascular fraction of adipose tissue constituted approximately 3% of the total cell population [10]. Specifically, BAC contains a high concentration of mitochondria and abundant capillaries for more oxygen and nutrients so as to generate more thermal energy [11]. Although representing a small proportion of adipose tissue, there is clinical evidence addressing the relationship between BAC and breast cancer [12,13]. The volume of BAC evaluated by positron emission tomography–computed tomography (PET/CT) was discovered as an independent predictive factor for tumor recurrence and mortality in cancer patients, regardless of the tumor subtype [14]. The BAC activity on FDG-PET/CT in breast cancer patients was three times higher than that in other solid tumor patients, and this difference is particularly significant among young women under the age of 55 [12]. Another study showed that higher BAC activity and volume were associated with an advanced stage of breast cancer [13]. However, the role of BAC within the breast cancer microenvironment remains unclear, especially for the interactive mechanisms between BAC and breast cancer cells.

With regard to the interaction between adipocytes and breast cancer cells, a more aggressive tumor microenvironment could be remodeled by the multipotent capabilities of ADSC, the pro-inflammatory property of WAC, and the thermogenic activity of BAC, resulting in altered cancer survival, proliferation, migration, angiogenesis, and metastasis. Particularly, it was reported that activated BAC could suppress breast tumor growth in animal models. Such an effect was achieved by reducing the source of glucose for the tumor by competing for blood glucose [15]. This controversial result from clinical findings suggests that the interaction between adipocytes and breast cancer was more complicated in the tumor microenvironment. Therefore, we aim to explore the effects of various adipocytes, particularly BAC, on luminal A subtype breast cancer invasiveness, and to investigate the direct or indirect interactions between breast cancer cells and different adipocytes, as well as to explore the underlying mechanisms at both cellular and molecular levels.

## 2. Materials and Methods

### 2.1. Cell Lines

Human luminal A breast cancer cells (MCF-7, ATCC^®^ HTB-22TM, American Type Culture Collection, Manassas, VA, USA) and human adipose-derived mesenchymal stem cells (ADSC, CP-H202, Procell Biotechnology Co., Ltd., Wuhan, China) were used in this study. All these cell lines passed mycoplasma screening and were validated by STR analysis.

### 2.2. Adipogenic Differentiation

The ADSC line (CP-H202) was used for differentiating into various types of adipocytes. Briefly, ADSC was induced by white adipocyte (WAC) induction medium (DMEM, 10% FBS, 0.5 mM IBMX, 1 μM Dexamethasone, 10 μg/mL Insulin, 1 μM Rosiglitazone) and maintained by maintenance medium (DMEM, 10% FBS, 10 μg/mL Insulin) for 14 days to obtain well-differentiated WAC. Brown adipocytes (BAC) were differentiated by induction using the WAC medium-based BAC medium added with 1 nM Triiodothyronine and 100 nM Ascorbic acid for 14 days, and maintained in the final BAC medium to obtain well-differentiated BAC.

### 2.3. Indirect Co-Culture System

MCF7 was cultured in the insert of a Transwell^®^ system (0.4 µm pore size; Corning Incorporated, Corning, NY, USA) in 6-well tissue culture plates (SPL Life Sciences Co., Ltd., Pocheon, Republic of Korea), and adipocytes were placed in the bottom. Initial cell count for both cell lines was 2 × 10^5^ cells to reach the ratio of 1:1. After 3 days of co-culture, breast cancer cells in the insert were collected in the pellets for further assays.

### 2.4. Direct Co-Culture System

MCF7 and adipocytes were mixed and co-cultured in a Transwell^®^ system for 3 days with their respective initial 2 × 10^5^ cells at a 1:1 ratio. For the co-cultures medium, a medium containing 50% MCF7 cell growth medium and 50% ADSC or mature adipocytes growth medium was used.

### 2.5. Cell Proliferation (MTT) Assay

Cells were seeded in a 96-well culture plate to reach the 80% confluence. The original culture medium was discarded, and 100 μL of serum-free culture medium containing 3% MTT was added. After incubation at 37 °C for 3–4 h, remove the medium and add 100 μL dimethyl sulfoxide (DMSO) to incubate at room temperature for 15 min. Finally, the optical density value (A570 value) was measured at 570 nm using a Microplate Photometer (BioTek, Inc., Winooski, VT, USA). All experiments were repeated three times.

### 2.6. Migration and Invasion Assays

For migration assays, Falcon^®^ Cell Culture Inserts (8 µm pore size; 24-well plate; Corning Incorporated, Corning, NY, USA) were used. For invasion assays, the assay was performed in 24-well BioCoat^®^ Matrigel^®^ Invasion Chambers (8 µm pore size; Corning Incorporated, Corning, NY, USA). For both assays, MCF7 cells were resuspended in serum-free DMEM and seeded into the pre-warmed cell culture inserts at a cell density of 2.5 × 10^5^ cells/well for Migration Assays) and 5 × 10^4^ cells/well for Invasion Assays, respectively. The bottom chambers contained either the same seeding density of adipocytes or regular growth medium (DMEM, 10% FBS, 1% AA). Upon 3 days of incubating at 37 °C, cell culture inserts were removed, and non-penetrated cells were removed from the upper side of the membrane using a cotton-tipped swab. The cells on the underside of the membrane were washed and fixed in 4% PFA and stained for 20 min in a crystal violet solution (0.5 mg/mL). The values for cell migration and invasion were calculated as the mean of migrated and invaded cells/field (total migrated/invaded cell number minus migrated/invaded adipocyte number). Normally, adipocytes do not invade through the membrane; therefore, the mean number of invaded cells/field was the definite cell counts in the lower chamber. Experiments were performed in duplicates and repeated at least three times with consistent results.

### 2.7. Fluorescent Staining

The slides with cell attachment were fixed with 4% paraformaldehyde for 15 min. Incubate the slides for 20 min with 0.25% Triton X-100 at RT. Drop blocking buffer onto a glass slide and seal it at room temperature for 30 min. Remove the blocking solution, add enough diluted primary antibody to each slide, and place it in a wet box. Then, incubate overnight at 4 °C. Discard the primary antibody solution and add diluted fluorescent-labeled secondary antibody. Incubate at 37 °C in a wet box for 1 h. Add DAPI and incubate in the dark for 5 min. Mount the coverslip with a drop of medium containing an anti-fluorescence quencher. Seal the coverslip and observe the slide under the microscope.

### 2.8. Quantitative Real-Time Polymerase Chain Reaction (qPCR)

Quantitative real-time PCR was performed with the Thermo Fisher Quantitative PCR machine (ABI 700; Thermo Fisher Scientific Inc., Waltham, MA, USA) according to the manufacturer’s instructions. The reaction of qRT-PCR went through an initial activation stage at 95 °C for 10 min, 45 cycles of three-step amplification, a denaturation stage at 95 °C for 10 s, an annealing stage at 60 °C for 30 s, and an extension stage at 72 °C for 30 s. Messenger RNA (mRNA) expression was normalized to reference gene expression and calculated by the 2^−∆∆Ct^ method (∆Ct = Ct_gene_ − Ct_ref_).

### 2.9. Western Blot

Purified protein samples were run on electrophoresis at 60 V for about 25 min, then 80 V for about 1.5 h to separate the protein. PVDF transmembrane was conducted at 90–110 V for about 1 h. After the membrane transfer was completed, the PVDF membrane was incubated on a shaker with 5% skim milk for 45 min. The PVDF membrane was incubated with the primary antibody overnight at 4 °C and then with the secondary antibody at room temperature for 1 h. ECL™ Prime Western blotting detection reagent (GE Healthcare UK Limited, Little Chalfont, Buckinghamshire, UK) was then applied to the membrane and visualized by the film.

### 2.10. Seahorse Mitochondrial Stress Assay

The Seahorse assay was performed according to the protocol provided by the manufacturer (Agilent Technologies, Inc., Santa Clara, CA, USA). Briefly, a 96-well plate is used for cell seeding, in which 80 μL pure Agilent Seahorse XF DMEM medium is added to 4 background wells and 80 μL DMEM medium containing around 5000–40,000 cells. The cell plate is then incubated at 37 °C overnight. The sensor cartridge is immersed in 200 μL of distilled water for hydration overnight at a 37 °C incubator without CO_2_. On the day of the experiment, the sensor cartridge plate is replaced with 200 μL Seahorse XF Calibrant for incubation at 37 °C without CO_2_ for another 45–60 min. Mito stress drugs such as Oligomycin, FCCP, and Rot/AA are prepared to reach the working concentrations at 15 μM, 2.5 μM, and 5 μM. After enough incubation at a 37 °C incubator without CO_2_, drugs are loaded into the 3 ports of the sensor cartridge. The sensor cartridge is then loaded into the Seahorse analyzer to initiate the test. Normalization should be conducted by adding 3 μL Hoechst 33342 to each well after the Seahorse analysis. The live cell number indicated by the Hoechst 33342 positive staining is used as the normalization factor. Result analysis is performed on the Seahorse XFe/XF analyzer and exported to GraphPad Prism 9 for figure configuration.

### 2.11. IRX3 Knockdown by siRNA

Knockdown of IRX3 is performed by transfecting IRX3 siRNA into the cells with the forward transfection procedure. Plate cells at 20 × 10^4^ cells per well in a 6-well culture plate and incubate for 24 h. Incubate the transfection master mix for 15 min, add it to the treatment well slowly, and incubate for 72 h. The knockdown efficiency is confirmed by qPCR and Western blot.

### 2.12. Clonogenic Assay

Dilute the cell suspension with gradient multiples, and plate 50, 100, and 400 cells with gradient densities in each dish containing 10 mL of 37 °C pre-warmed culture medium. Gently shake to disperse the cells evenly. Change the medium and observe the cell status every 3 days midway, and terminate the culture when the number of cells in the vast majority of individual clones exceeds 50. After cloning is completed, take photos of the cells under a microscope and wash them with PBS once. Add 4% paraformaldehyde to each sample and fix for 30 min. Wash the cells with PBS once. Add an appropriate amount of crystal violet staining solution to each sample and stain the cells for 10–20 min. Wash the cells several times with PBS, air dry, and take photos. No treatment and scrambled siRNA are used as the controls.

### 2.13. Orthotopic Breast Cancer Xenograft Mouse Model

NOD-SCID female mice at 4 weeks of age were used for the model establishment. The ethical approval was obtained before conducting the animal studies. The mice were supplied with sterilized water and standard laboratory chow diet and were kept in individual ventilated cages with a 12 h light/12 h dark cycle. Briefly, after successful anesthesia, 1 × 10^6^ cells suspended in 100 μL PBS containing 50% Matrigel (BD Biosciences Discovery Labware, Bedford, MA, USA) were inoculated subcutaneously near the fourth mammary fat pad of mice. One week after cell implantation, each mouse was started to receive intraperitoneal injection with 0.2 mg/kg 17 β-estradiol and continuously injected twice a week until the endpoint of the experiment. The size of the tumors was determined by measuring the largest and smallest tumor diameter (Length, Width) every week throughout the experiment and was calculated as Volume = Length × Width^2^ × 0.52. The endpoint event was set as the maximum diameter of the tumor reaching 1.5 cm or death. At the end of the animal study, an overdose of pentobarbital was administered to sacrifice the mice. The weight and the volume of xenograft tumors were measured and photographed. Fresh tumor tissues were frozen at −80 °C or fixed with formalin for future study.

### 2.14. Immunohistochemical Staining

Perform heat-mediated antigen retrieval with Tris/EDTA buffer (PH 9.0) in the microwave at 65 °C for 20 min. PBS is washed 3 times. Add one drop of 3% H_2_O_2_ to each slide and incubate at room temperature in the dark for 10 min to block the activity of endogenous peroxidase. Incubate the slides with primary antibody in a 37 °C black box for 1 h. Rinse with PBS three times, immerse slides in trisTBS for 6 min. Add secondary antibody to the shaking bed at 37 °C for 25 min. Prepare the mix of chromogen and H_2_O_2_ at a ratio of 1:1, cover the mix on slides for 8 min. Immerse the slides in Gill’s Hx for 5 min and put them into Scot’s water for 1 min. Stain the slides with hematoxylin and seal the slides with mounting medium. Images are acquired under a microscope using bright-field illumination.

### 2.15. Statistics Analysis

All animal and in vitro data are presented as means and standard errors. The comparison between different groups is conducted by an unpaired Student’s *t*-test. The statistical significance is determined when the *p*-value of 0.05 is reached. All in vitro experiments are repeated at least three times. Quantifications for images are analyzed by ImageJ (ImageJ 1.53a, Wayne Rasband, National Institutes of Health, USA). GraphPad Prism 9.0 is used for data analysis.

## 3. Results

### 3.1. Mature Adipocytes Exert Promoting Effects on Luminal A Breast Cancer Invasiveness

**Figure 1:**
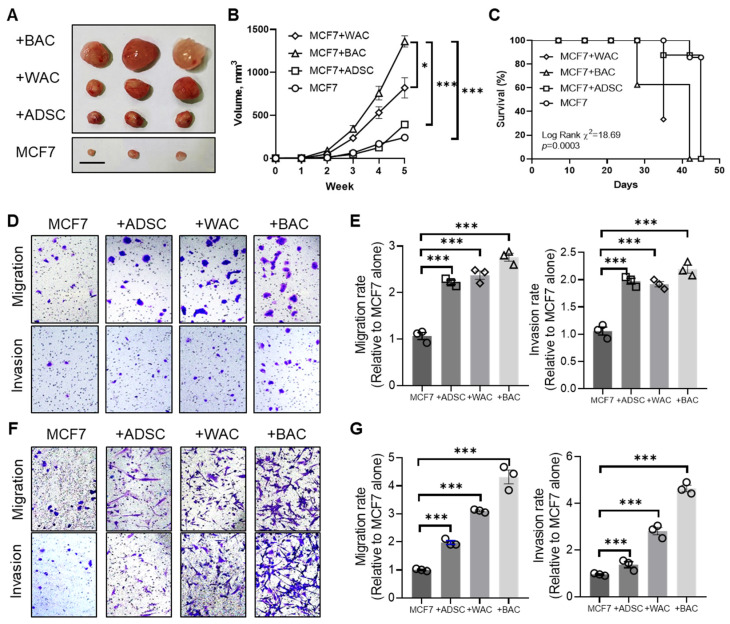
Adipocytes promote breast cancer cell invasiveness. (**A**) Orthotopic tumor comparison among groups (*n* = 3 each group), scale bar indicates 10 mm; BAC, brown adipocyte; WAC, white adipocyte; ADSC, adipose-derived mesenchymal stem cell. (**B**) Tumor growth curve for all tumors injected into the subcutaneous space of the fourth pad of an adult female mouse (*n* = 5 each group). The Y-axis indicates tumor volume in mm^3^. *, *p* < 0.05, ***, *p* < 0.001. (**C**) Overall survival curve for all mice injected with breast cancer cells and adipocyte mixture. (**D**) Representative images of transwell assays show the migration and invasion abilities of indirect co-cultures. (**E**) Quantification of relative migration and invasion (*n* = 3). *** *p* < 0.001. (**F**) Representative images of migrated and invaded cells among direct co-culture systems in the transwell system; representative images of adipocyte-only migration are also shown. (**G**) Quantification of cell migration and invasion of breast cancer cells from the insert of the transwell systems. ***, *p* < 0.001.

**Figure A1:**
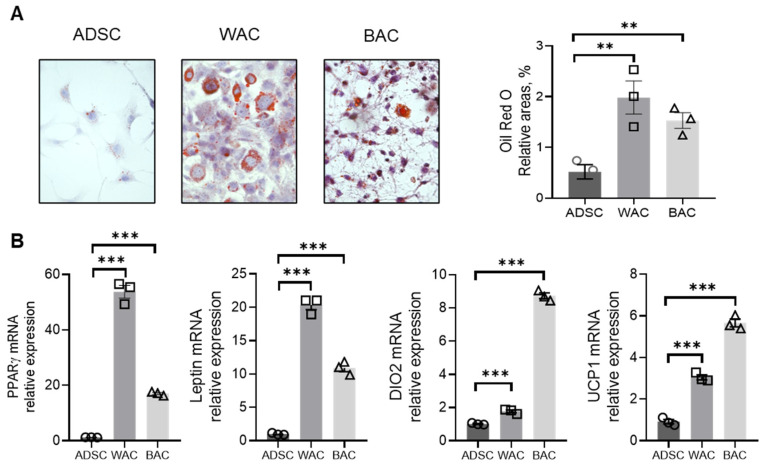
Differentiation into mature adipocytes from adipose-derived mesenchymal stem cells (ADSC). (**A**) Lipid droplet formation in ADSC, WAC, and BAC is indicated by oil red O staining. Quantification results for oil red O staining images were obtained among different adipocytes. (**B**) Real-time qPCR for PPARγ, leptin, DIO2, and UCP1 for validation of adipocytes after differentiation. All data are fold change in mRNA expression relative to the reference gene GAPDH. **, *p* < 0.01; ***, *p* < 0.001.

The effects of various types of adipocytes on breast cancer cell behavior were studied in an orthotopic breast cancer xenograft mouse model. To prepare the mature adipocytes for orthotopic transplantation, white adipocyte (WAC) and brown adipocyte (BAC) were successfully differentiated from adipose-derived mesenchymal stem cells (ADSC), and their phenotypes were validated by lipid formation and subtype-specific marker expression (Figure A1). Then, the luminal A breast cancer cell line, MCF7, and various types of adipocytes were mixed together at a ratio of 1:1 before transplantation. To investigate the impact on breast cancer invasiveness exerted by adipocytes, an orthotopic breast tumor xenograft mouse model was established in NOD-SCID mice. After 5-week transplantation, the average tumor size was nearly 6 times larger in the MCF7+BAC group when compared to the MCF7 alone, and was 1.2–4 times larger than that in the MCF7+WAC and MCF7+ADSC groups (Figure 1A). After longitudinal observations, the tumor growth rate was significantly higher in the MCF7+BAC group than in the other mixture groups and the MCF7 alone group (Figure 1B). As the long-term outcome, the overall survival of mice in the MCF7+BAC group was lower than that in the other groups (Figure 1C).

Indirect co-culture between MCF7 and adipocytes was performed by placing MCF7 and adipocytes in the insert and lower chamber of the transwell, respectively (Figure 1D). The result showed a significant increase in cell migration and invasion when compared to MCF7 alone (Figure 1E). The direct co-culture of MCF7 and various types of adipocytes was also conducted in transwell systems. Direct co-cultivation of MCF7 and adipocytes showed higher rates of migration and invasion (Figure 1F). Notably, the most prominent effects were observed in the BAC group, which increased MCF7 invasiveness by five-fold compared to MCF7 alone, whereas the least effects were seen in the ADSC group (Figure 1G). The above data suggested that direct contact of adipocytes with breast cancer would exert more effects than indirect co-culture, among which the brown adipocyte was the most prominent player.

### 3.2. Brown Adipocyte Promoted Luminal A Breast Cancer Invasiveness

**Figure 2:**
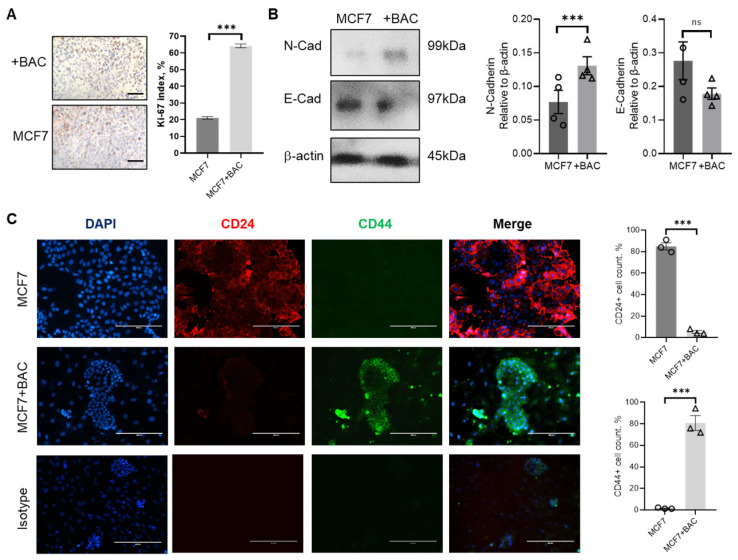
Brown adipocyte promoted luminal A breast cancer cell proliferation and stemness. (**A**) Representative immunohistochemistry staining for tumor proliferation analysis. Comparison was between MCF7 alone and the co-cultured cell mixture. Scale bar indicates 100 μm. The bar chart indicates the Ki-67 index among groups. ***, *p* < 0.001. (**B**) Representative Western blot images for N-Cadherin and E-Cadherin for MCF7 and BAC co-culture and MCF7 alone (n = 4 in each group). The bar chart indicates the quantification. ns, not significant; ***, *p* < 0.001. (**C**) Immunofluorescence images showing the expression of CD44 and CD24 in MCF7+BAC and MCF7 alone. Cells are labeled with anti-CD44-488, anti-CD24-PE, and the nuclei stain Hoechst. Isotype staining is set as the control. The bar chart indicates the quantifications for CD24+ and CD44+ cell percentages among all cells. Scale bar: 20 μm.

Brown adipocyte was further investigated regarding the tumor proliferation, epithelial–mesenchymal transition (EMT), and cancer stemness. Regarding the tumor behavior, immunohistochemistry staining for Ki-67 suggested that the proliferation of the tumor in the group of MCF7+BAC was enhanced compared to that in MCF7 alone, and the quantification of the Ki-67 index also showed significant differences (Figure 2A). EMT was also up-regulated after co-culturing with BAC, as the immune blots showed that N-Cadherin was increased but E-Cadherin was decreased (Figure 2B). Furthermore, enhanced cell invasiveness was investigated regarding the stemness. By comparison with MCF7 alone, the ratio of cancer stem cells indicated by CD44^+^CD24^−^ was significantly higher in the co-culture of MCF7+BAC (Figure 2C).

### 3.3. IRX3-Related Mitochondrial Dysfunction Was Observed as a Molecular Signature in Brown Adipocyte Promoting Breast Cancer Invasiveness

**Figure 3:**
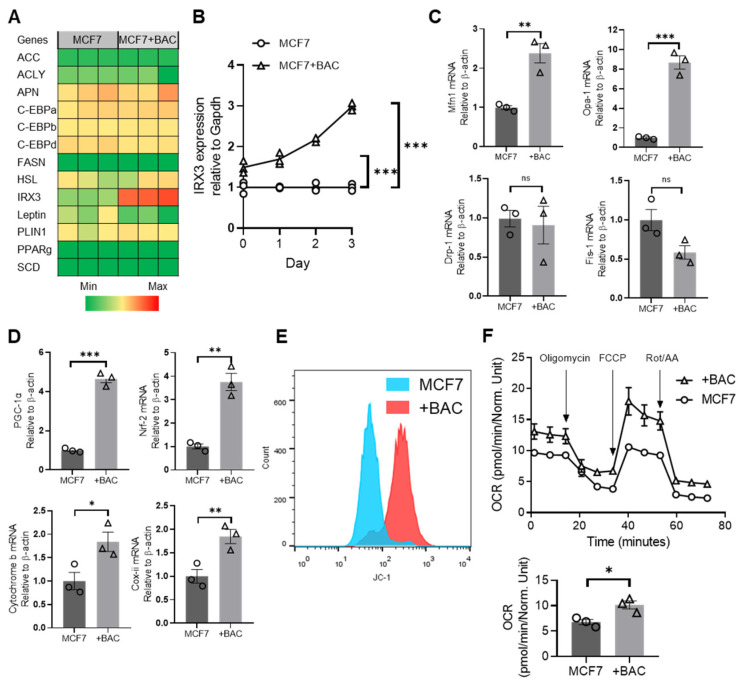
Brown adipocytes exerted the effects on MCF7 via IRX3-related adipogenesis and mitochondrial dysregulation. (**A**) Heatmap for adipogenesis-related genes’ transcription. Comparison between MCF7+BAC co-culture and MCF7 alone. (**B**) Longitudinal transcriptional levels of IRX3 in MCF7, and fusion hybrids of MCF7+BAT or MCF7+ASC for continuous 3-day co-culture. Longitudinal protein levels of IRX3 in MCF7+BAC co-culture and MCF7 alone. (**C**) Transcript levels of mitochondrial fusion-related genes (Mfn1, Opa-1) and fission-related genes (Drp-1, Fis-1) between MCF7 and MCF7+BAC co-culture. (**D**) Transcript levels of mitochondrial biogenesis-related genes (PGC-1α, Nrf-2) and mitochondrial respiratory chain genes (cytochrome b, cytochrome c oxidase subunit 2 (Cox-ii)) between MCF7 and MCF7+BAC co-culture. (**E**) Mitochondrial membrane potential change indicated by JC-1 staining in MCF7 and MCF7+BAC co-culture. (**F**) Oxygen consumption rate comparison between MCF7 and MCF7+BAC co-culture. Dots represent means of oxygen consumption rate (pmol/min), error bars indicate standard error. ns, no significance; ns, not significant; * *p* < 0.05; **, *p* < 0.01; ***, *p* < 0.001.

To investigate the molecular mechanisms of enhanced cancer invasiveness by brown adipocytes, the expression of adipogenesis-related genes was explored by quantitative PCR. The result showed that, when normalized to the expression of genes in MCF7 cells, the transcriptional level of IRX3 in MCF7 and BAC co-culture was significantly higher than that of MCF7 alone, among all genes related to adipogenesis (Figure 3A). Additionally, the longitudinal expression levels of IRX3 in the MCF7+BAC group were continuously increased compared with the MCF7 group (Figure 3B). As adipogenesis always resulted in altered metabolism, we further studied the expression levels of mitochondrial function-related genes and found that the mitochondrial dynamics and bioenergetics changed significantly after co-culturing with BAC. Genes related to mitochondrial fusion were elevated, but genes for mitochondrial fission were decreased in the MCF7+BAC group (Figure 3C). In addition, transcriptional expressions of mitochondrial biogenesis and oxidative phosphorylation were significantly higher in the MCF7+BAC group (Figure 3D). The mitochondrial functional alteration was also demonstrated by the increased mitochondrial viability indicated by membrane potential signals in the MCF7+BAC group (Figure 3E). Consequently, the mitochondrial function examination showed that the maximal respiration of the MCF7+BAC group was significantly higher than that of MCF7 alone (Figure 3F). The results indicate that the upregulated IRX3 and dysregulated mitochondrial function in the scenario of BAC promoting cancer invasiveness warrant further investigation.

### 3.4. IRX3 Mediated Mitochondrial Regulation in Luminal A Breast Cancer

**Figure 4:**
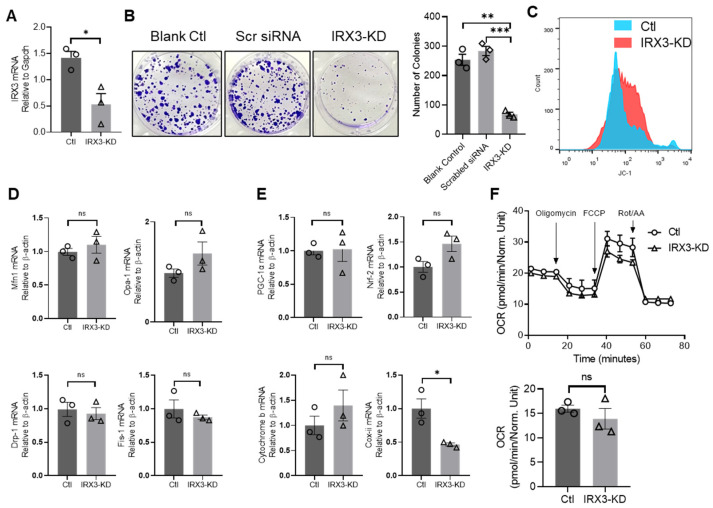
IRX3 mediated the mitochondrial dysregulation during brown adipocyte–breast cancer interaction. (**A**) The bar chart represents the relative IRX3 mRNA levels in MCF7 and MCF7 with IRX3 knockdown. (**B**) Representative images and quantification of colony formation for MCF7 with no treatment (blank control), scrambled siRNA, and IRX3 siRNA knockdown are shown. The bar chart represents the number of colonies. (**C**) Mitochondrial membrane potential change indicated by JC-1 staining in MCF7+BAC co-cultures without (Ctl) and with IRX3 knockdown. (**D**) Transcript levels of mitochondrial fusion-related genes (Mfn1, Opa-1) and fission-related genes (Drp-1, Fis-1) in MCF7+BAC co-cultures without (Ctl) and with IRX3 knockdown. (**E**) Transcript levels of mitochondrial biogenesis-related genes (PGC-1α, Nrf-2) and mitochondrial respiratory chain genes (cytochrome b, cytochrome c oxidase subunit 2 (Cox-ii)) in MCF7+BAC co-cultures without (Ctl) and with IRX3 knockdown. (**F**) Oxygen consumption rate comparison in MCF7+BAC co-cultures without (Ctl) and with IRX3 knockdown. Dots represent means of oxygen consumption rate (pmol/min), error bars indicate standard error. ns, no significance; ns, not significant; * *p* < 0.05; **, *p* < 0.01; ***, *p* < 0.001.

Given the critical upregulation of IRX3 in MCF7 after co-culture with BAC, we then knocked down IRX3 in MCF7 cells and explored the role of IRX3 in breast cancer invasiveness. After IRX3 knockdown, the IRX3 mRNA level was significantly down-regulated (Figure 4A). Consistently, the number of colonies formed by MCF7 single cells decreased (Figure 4B). Correspondingly, the mitochondrial membrane potential was increased after IRX3 knockdown, indicated by increased JC-1 staining (Figure 4C). The mitochondrial dysfunction regarding dynamics and bioenergetics was ameliorated after IRX3-knockdown MCF7 cells were co-cultured with BAC again (Figure 4D,E). As a result, mitochondrial oxygen consumption rate was reduced to a similar level as MCF7+BAC co-culture without IRX3 manipulation (Figure 4F). The above data suggest that it was IRX3 upregulation that regulated mitochondrial dysfunction during BAC, exerting effects on MCF7, and the dysfunction could be ameliorated after IRX3 knockdown.

### 3.5. IRX3 Knockdown Reduced Luminal A Breast Cancer Invasiveness

**Figure 5:**
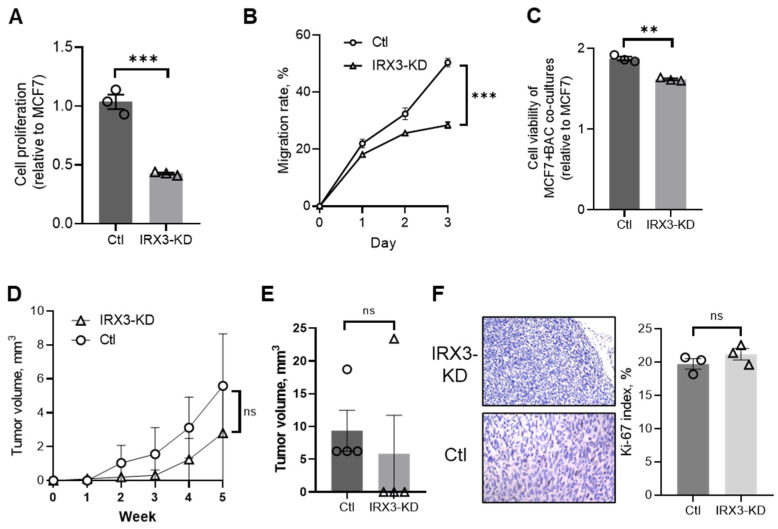
IRX3 knockdown ameliorated breast cancer invasiveness in vitro and in an animal model. (**A**) Cell proliferation rate indicated by MTT assay for MCF7 without treatment and MCF7 after IRX3 knockdown. (**B**) Cell migration indicated by the wound healing assay. Quantifications for wound healing longitudinal were shown. (**C**) Cell proliferation rate indicated by MTT assay in MCF7 alone (Ctl) and MCF7+BAC co-culture with IRX3 knockdown. (**D**) Tumor growth curve for MCF7+BAC co-culture without IRX3 knockdown (Ctl) and MCF7+BAC co-culture with IRX3 knockdown implantation. (**E**) Quantification for the final tumor volume at 5 weeks, injected with MCF7+BAC co-culture without IRX3 knockdown (Ctl) and MCF7+BAC co-culture with IRX3 knockdown. (**F**) Representative immunohistochemistry staining for tumor proliferation analysis. Comparison was between orthotopic tumors injected with MCF7+BAC co-culture without IRX3 knockdown (Ctl) and MCF7+BAC co-culture with IRX3 knockdown. The bar chart indicates the Ki-67 index between groups. ns, no significance; **, *p* < 0.01; ***, *p* < 0.001.

In vitro data showed that MCF7 with IRX3 knockdown reduced the proliferation rate (Figure 5A). In addition, the wound healing assay showed that IRX3 knockdown significantly slowed down the cell migration rate (Figure 5B). In the setting of co-culture, MCF7 with IRX3 knockdown was resistant to BAC-promoted cancer cell survival (Figure 5C).

We further implanted co-cultured cells between BAC and MCF7 with IRX3 knockdown into NOD-SCID mice and compared the behavior with MCF7 implantation alone. As a result, the longitudinal observations showed that the mixture of BAC and MCF7 with IRX3 knockdown exhibited no significant advantage over MCF7 alone, and the difference in final tumor size validated this growth trend (Figure 5D,E). By pathological examination, the Ki-67 index of the MCF7+BAC group with IRX3 knockdown showed no significant difference compared with the MCF7-alone tumor, which is consistent with cell mixture inoculation results (Figure 5F).

## 4. Discussion

In this study, we demonstrated that distinct types of adipocytes enhance breast cancer invasiveness, irrespective of whether the effects are mediated directly or indirectly. Despite numerous previous reports that have investigated the impact of specific adipocyte subtypes on breast cancer, this study represents the first comprehensive analysis of the effects of various adipocytes on breast cancer [15,16,17]. Through molecular dissection, we identified dysregulated intracellular adipogenesis and mitochondrial dysfunction as the underlying mechanisms, with IRX3-mediated mitochondrial dysfunction as a key molecular pathway that may serve as a potential therapeutic target.

The effect of adipocyte-promoting breast cancer invasiveness was particularly significant in BAC-co-cultured MCF7 cells. This finding was further consolidated in an orthotopic xenograft mouse model of breast cancer. Interestingly, although ADSCs have been implicated as potential drivers of cancer recurrence after breast reconstruction, no significant tumor growth was observed when ADSCs were mixed with MCF7 cells. These in vitro and in vivo phenotypes suggest that ADSC-added lipofilling is relatively safe, whereas attention should be given to a minor adipocyte population, such as BAC. Accumulating evidence indicates that enhancing the functional quality of BAC by promoting white adipose tissue (WAT) browning may contribute to combating obesity and diabetes [18]. Moreover, studies have suggested that BAC may improve the retention of autologous fat grafts by inhibiting the Wnt/β-Catenin pathway [19] and that lipofilling can induce the browning of WAT [20]. Although common suction sites for lipoaspirates, such as subcutaneous adipose tissue from the abdomen wall, buttocks, and thighs, are predominantly composed of WAT [21], the relative proportions of BAC, ADSCs, and other adipocyte subtypes within the fat grafts remain unknown. Furthermore, metabolic changes in adipose tissue after transplantation to a new anatomical site may influence adipose characteristics. Nevertheless, our experimental data indicate that BAC is the most hazardous subtype among all adipocytes within the breast cancer microenvironment. Therefore, risk assessment of lipofilling should be carefully reconsidered due to the presence of BAC and the potential for WAT browning.

Consistent with the observed increase in invasiveness, we found a parallel enhancement in adipogenesis, upregulation of IRX3, and increased mitochondrial respiratory function. The role of adipogenesis in breast cancer has received widespread attention due to its significant contribution to cancer occurrence, progression, recurrence, and metastasis. The mechanism through which adipogenesis influences breast cancer invasiveness involves cellular and molecular interactions within the tumor microenvironment (TME) [22]. Current studies indicate that breast cancer cells can exhibit “adipogenicity phenotype” and that the degree of adipogenicity varies among breast cancer molecular subtypes in terms of fatty acid sensitivity and lipid metabolism [23]. Moreover, adipose tissue–breast cancer crosstalk has been shown to enhance de novo adipogenesis and lipid metabolism in cancer cells, further remodeling the TME [24]. The mechanisms underlying enhanced tumor adipogenesis are multifactorial. In addition to direct lipid transfer into tumor cells during direct interaction, the specific adipogenesis-related molecules can be initiated or upregulated upon interaction with other cell types. These regulatory molecules include key enzymes involved in fatty acid synthesis (FASN, ACLY, ACC, SCD, HSL), lipid droplet breakdown proteins (PLIN1, etc.), fatty acid transport and storage proteins (FABP4, etc.), members of the C/EBP family, and transcription factors such as PPARγ and IRX3 [25]. Notably, IRX3 was the most prominently upregulated molecule in the highly invasive MCF7+BAC group. Increasing evidence suggests that IRX3 plays an essential role in adipose biology and metabolism, added by other transcription factors to regulate the transcription of genes involved in lipid synthesis, fatty acid metabolism, and adipocyte function [26]. Aberrant expression of IRX3 may lead to alterations in adipocyte number and distribution, thereby affecting body fat accumulation and distribution [27]. Although obesity is an independent risk factor for breast cancer, the oncogenic effects of IRX3 on breast cancer invasiveness and whether these effects are independent of obesity remain unknown. In this study, we investigated the biological behavior of HR+ MCF7 cells before and after IRX3 knockdown. IRX3 knockdown resulted in decreased proliferation and migration of MCF7 cells. These effects were also observed in the co-culture of IRX3-knockdown MCF7 cells with adipocytes. Although the role of IRX3 in other breast cancer molecular subtypes requires further investigation and validation, this study is the first to establish an association between IRX3 and breast cancer invasiveness, which potentially provides new therapeutic insights for breast cancer treatment.

Lastly, given the differences in mitochondrial content among different adipocytes, we sought to study whether differences in mitochondrial function, such as energy metabolism, fusion and fission, and biogenesis, between breast cancer cells and co-cultures exist. Our findings revealed that mitochondrial oxygen consumption rate was significantly enhanced in MCF7+BAC co-culture compared to MCF7 cells alone, indicating the enhanced mitochondrial oxidative phosphorylation (OXPHOS). This finding is consistent with the observation that the MCF7+BAC co-culture exhibits the highest tumor invasiveness. Under normal physiological conditions, non-malignant cells primarily rely on OXPHOS to produce ATP, whereas cancer cells typically utilize glycolysis as the source of ATP production (Warburg effect). However, our results show that the co-culture cells exhibit enhanced OXPHOS, which is uncommon in malignant cells. Theoretically, co-culture may facilitate the transfer of substance or bioenergy to recipient cells, allowing the recipient cancer cell to exhibit the preference to OXPHOS because of its inheritance from adipocytes [28]. Therefore, enhanced mitochondrial metabolic activity in tumor cells implies their ability to produce and supply energy more efficiently to meet the high energy demands of rapid division and growth. Moreover, enhanced mitochondrial metabolic activity may equip tumor cells to adapt to harsh microenvironmental conditions, such as hypoxia and nutrient deprivation in the spheroid-like structures, thereby improving their survival and adaptability [29].

## 5. Conclusions

The breast cancer microenvironment was significantly impacted by various types of adipocytes, among which brown adipocytes exerted the most prominent effects on cancer invasiveness. The enhanced invasiveness by brown adipocytes was mediated by IRX3-related adipogenesis, increased mitochondrial biogenesis and bioenergetics, and acquisition of cancer stemness. The altered molecular and cellular mechanisms potentialize the development of new targeted therapy, cell therapy for breast cancer treatment, as well as new biomarkers for precisely predicting outcomes.

## Data Availability

All data presented in this study are available upon request to the corresponding author.
